# Alarming Rise in Untreatable Neonatal Sepsis: A Prospective Study of Multidrug-Resistant (MDR) and Extensively Drug-Resistant (XDR) Pathogens in an Indian Tertiary NICU

**DOI:** 10.7759/cureus.108753

**Published:** 2026-05-12

**Authors:** Ravi Kumar, Soumini Rath, Subhra Snigdha Panda, Manas K Nayak, Nirmal K Mohakud

**Affiliations:** 1 Pediatrics, Kalinga Institute of Medical Sciences, Bhubaneswar, IND; 2 Pediatrics, Lord Buddha Koshi Medical College and Hospital, Saharsa, IND; 3 Neonatology, Kalinga Institute of Medical Sciences, Bhubaneswar, IND; 4 Medical Microbiology, Kalinga Institute of Medical Sciences, Bhubaneswar, IND; 5 Pediatric Medicine, Kalinga Institute of Medical Sciences, Bhubaneswar, IND

**Keywords:** antimicrobial resistance, extensively drug-resistant, multidrug resistant, neonate, nicu, sepsis

## Abstract

Background: Culture-positive neonatal sepsis is a leading cause of mortality. The emergence of multidrug-resistant (MDR) and extensively drug-resistant (XDR) bacteria exacerbates the clinical outcomes. This study aimed to determine the bacteriological profile and antibiotic resistance patterns of MDR and XDR strains causing neonatal sepsis.

Objectives: This study aimed to determine the prevalence of culture-positive neonatal sepsis, characterize the bacteriological profile and antimicrobial resistance patterns of multidrug-resistant (MDR) and extensively drug-resistant (XDR) pathogens, and assess clinical outcomes, specifically mortality, in neonates admitted to a tertiary care NICU.

Methods: A prospective observational study was conducted from August 2020 to September 2022. Neonates with clinical signs of sepsis and positive blood cultures were enrolled. Bacterial identification and antibiotic susceptibility testing were performed using the VITEK 2 compact automated system (Marcy-l'Étoile, France: bioMérieux). Isolates were classified as MDR or XDR based on standardized international definitions.

Results: Of 120 culture-positive neonates, 100 (83.3%) had bacterial sepsis. Gram-negative bacteria accounted for 67 of 100 bacterial isolates (67%); the most common pathogens were *Klebsiella pneumoniae* (19/67; 28.4%) and Acinetobacter species (10/67; 14.9%). Overall, 55 of 100 bacterial isolates (55.0%) were multidrug-resistant (MDR), and 19 of 100 (19.0%) were extensively drug-resistant (XDR). High resistance rates were observed for fluoroquinolones (59/98 tested; 60.2%), carbapenems (30/59 tested; 50.8%), and third-generation cephalosporins among Gram-negative isolates (42/66 tested; 63.6%). Among XDR isolates, resistance was 13/14 (92.9%) to piperacillin-tazobactam and 16/17 (94.1%) to cephalosporins. Mortality was higher in XDR cases (6/19; 31.6%) than in MDR cases (7/55; 12.7%).

Conclusion: There is a high burden of MDR and XDR Gram-negative neonatal sepsis, predominantly due to *K. pneumoniae* and Acinetobacter species. The alarming resistance to last-resort antibiotics underscores the critical need for robust antibiotic stewardship programs and continuous surveillance to prevent the emergence of pan-drug-resistant strains.

## Introduction

Neonatal sepsis remains a formidable global health challenge, contributing to approximately one-third of neonatal mortality in India [1]. In low- and middle-income countries in particular, neonatal sepsis-attributable mortality rates range from 10% to 29%, and it is recently estimated that sepsis causes 35% of neonatal deaths among the 2.4 million deaths occurring annually [2]. The case fatality rate escalates significantly with culture-positive sepsis, ranging from 33% to 55% [3]. This dire situation is compounded by the relentless rise of antimicrobial resistance (AMR). Multidrug-resistant (MDR) organisms, defined as non-susceptibility to at least one agent in three or more antimicrobial categories, and extensively drug-resistant (XDR) organisms, defined as non-susceptibility to at least one agent in all but two or fewer categories, are associated with higher treatment failure and mortality rates [4].

The genesis of AMR is a complex evolutionary process driven by selective antibiotic pressure, leading to the acquisition and dissemination of resistance genes via plasmids, transposons, and clonal expansion of high-risk bacterial lineages [5]. Among these, *Klebsiella pneumoniae *and Acinetobacter species have emerged as notorious pathogens in neonatal intensive care units (NICUs) worldwide, often harboring resistance to broad-spectrum beta-lactams, fluoroquinolones, and aminoglycosides [6,7]. Their ability to cause outbreaks and persist in hospital environments makes them particularly threatening [8].

India currently accounts for nearly one-fifth of global neonatal deaths attributed to sepsis [9]. Recent national surveillance data highlight a deteriorating antimicrobial resistance landscape; for instance, the 2024 Indian Council of Medical Research (ICMR) AMR report indicates that carbapenem susceptibility in *Klebsiella pneumoniae* has plummeted to approximately 31-35%, while susceptibility to piperacillin-tazobactam remains at 26% [10]. Furthermore, recent multi-center and regional studies report that multidrug-resistant (MDR) phenotypes are present in over 80% of Acinetobacter spp. isolates, with extensively drug-resistant (XDR) strains now accounting for nearly 38.4% of all Gram-negative neonatal sepsis cases, carrying a significantly higher mortality rate of up to 46.7% compared to drug-sensitive infections [11,12].

This study was conducted at a tertiary care NICU in Odisha, India, to delineate the contemporary bacteriological profile and antibiotic sensitivity patterns of MDR and XDR neonatal sepsis. In this study, "untreatable" neonatal sepsis refers primarily to infections caused by extensively drug-resistant (XDR) pathogens, which are non-susceptible to nearly all available antibiotic classes, and pan-drug-resistant (PDR) strains, for which no effective antimicrobial options remain in the standard local formulary. By characterizing the prevailing pathogens and their resistance mechanisms, this research aimed to provide evidence essential for optimizing treatment protocols and mitigating the threat of untreatable neonatal infections.

## Materials and methods

Study design and setting

This prospective observational study was conducted in the NICU of Kalinga Institute of Medical Sciences (KIMS), Bhubaneswar, Odisha, from August 2020 to September 2022. The study protocol was approved by the Institutional Ethics Committee (#KIIT/KIMS/IEC/473/2020), and consent was obtained from the parents for the anonymized use of laboratory and clinical data.

Study participants

All inborn and outborn neonates (0-28 days) admitted to the NICU with clinical suspicion of sepsis, identified by signs such as lethargy, respiratory distress, and poor perfusion, or by perinatal risk factors such as maternal fever (>38°C) and prolonged rupture of membranes (PROM) lasting more than 24 h, were screened [13]. Neonates with major congenital anomalies or those whose parents did not provide consent were excluded. Cultures identifying common skin contaminants (e.g., coagulase-negative Staphylococci, Bacillus spp., or Diphtheroids) were excluded. To ensure diagnostic accuracy, venous blood samples were collected from all patients before the initiation of empirical antibiotic therapy using strict aseptic techniques. Sepsis was strictly classified into the following two categories based on the timing of symptom onset: early-onset sepsis (EOS) was defined as clinical signs and a positive blood culture occurring within the first 72 h of life, while late-onset sepsis (LOS) was defined as onset after 72 h and up to 28 days, aligning with the National Neonatology Forum (NNF) India guidelines.

The study utilized a time-bound, prospective census approach. The study population was drawn from 1,126 neonates representing the total admissions to the KIMS NICU during the 25-month study period. Of these admissions, 120 neonates met the specific inclusion criteria (clinical suspicion followed by a confirmed positive blood culture) and were enrolled for detailed microbiological analysis. We utilized consecutive sampling of all eligible cases over the study duration to minimize selection bias and to capture the full spectrum of disease severity. The final sample size of 120 was not pre-determined by a power calculation but was dictated by the natural occurrence of culture-proven sepsis within the defined study window, providing a representative denominator for calculating the unit-specific prevalence of MDR and XDR pathogens.

The Kaiser Permanente neonatal sepsis calculator was not utilized for risk stratification in this study. This decision was based on the fact that the calculator is primarily validated for the management of asymptomatic infants born at ≥34 weeks' gestation. As our study population consisted of symptomatic neonates across all gestational ages, including a significant number of outborn referrals with incomplete maternal data, we prioritized clinical suspicion and National Neonatology Forum (NNF) India guidelines to determine the need for blood cultures and empirical therapy.

Sample collection and microbiological processing

Blood (1.5-2 mL) samples were collected under aseptic conditions from neonates with suspected sepsis, preferably before initiation of antibiotic therapy. Samples were inoculated into BacT/ALERT PF Plus (Durham, NC: bioMérieux) bottles and processed using the automated BacT/ALERT 3D system (Marcy-l'Étoile, France: bioMérieux). Cultures were incubated for up to five days or until flagged positive. Positive samples were subcultured onto blood agar and MacConkey agar, then incubated at 37°C for 24 h.

Bacterial identification and antimicrobial susceptibility testing

Identification and antimicrobial susceptibility testing were performed using the VITEK 2 compact system (Marcy-l'Étoile, France: bioMérieux). Results were interpreted according to Clinical and Laboratory Standards Institute (CLSI) guidelines [14]. Multidrug resistance (MDR) was defined as non-susceptibility to at least one agent in three or more antimicrobial classes [4]*. *Fungal isolates were identified using the VITEK 2 compact system (using the VITEK 2 yeast ID card) and appropriate mycological culture media (Sabouraud dextrose agar).

For Gram-negative bacteria (GNB), antimicrobial susceptibility testing included aminoglycosides (gentamicin, amikacin), piperacillin-tazobactam, third- and fourth-generation cephalosporins (ceftazidime, cefotaxime, cefepime), carbapenems (imipenem, meropenem), fluoroquinolones (ciprofloxacin), tigecycline, trimethoprim-sulfamethoxazole, and colistin. For Gram-positive bacteria (GPB), the panel included vancomycin, teicoplanin, linezolid, tetracycline, aminoglycosides, fluoroquinolones, carbapenems, and trimethoprim-sulfamethoxazole.

Data collection and analysis

Demographic and clinical data (birth weight, gestation, mode of delivery, clinical features, treatment, and outcome) were recorded on a pre-designed proforma. Microbiological data (organism identified, antimicrobial susceptibility testing (AST) profile, MDR/XDR status) were collated. Descriptive statistics (frequency, percentage, mean/median) were used for data analysis using SPSS Statistics version 25 (IBM Corp: Armonk, NY).

## Results

During the study period, there were 1,126 NICU admissions. Blood culture was positive in 120 neonates. Of these, 100 (83.3%) had bacterial sepsis, and 20 (16.7%) had fungal sepsis. Among the 100 bacterial isolates, 67 (67%) were Gram-negative bacteria (GNB), and 33 (33%) were Gram-positive cocci (GPC). Overall, 55 isolates (55%) were MDR, and 19 (19%) were XDR.

Among the 67 Gram-negative bacterial isolates, *Klebsiella pneumoniae* was the predominant pathogen (28.4%), followed by Acinetobacter species (14.9%) and *Burkholderia cepacia* (11.9%). Nearly half of these Gram-negative isolates (47.7%) were classified as multidrug-resistant (MDR), and a quarter (25.4%) were extensively drug-resistant (XDR). *Klebsiella pneumoniae* was also the most frequent organism within both the MDR (28.1% of MDR strains) and XDR (29.4% of XDR strains) categories, underscoring its central role in resistant neonatal sepsis in this setting (Table 1). Gram-positive bacteria accounted for 33 (33%) of the 100 bacterial isolates. *Staphylococcus aureus* was the most prevalent pathogen (13, 39.4%), followed by coagulase-negative Staphylococci (CoNS) (10, 30.3%) and Enterococcus spp. (7, 21.2%) (Table 2).

**Table 1:** Distribution of multidrug-resistant and extensively drug-resistant Gram-negative bacteria (n=67). MDR: multidrug-resistant; XDR: extensively drug-resistant

Organism	Total	MDR	XDR
*Klebsiella pneumoniae*, n (%)	19 (28.35)	9 (28.12)	5 (29.41)
Acinetobacter species, n (%)	10 (14.92)	4 (12.5)	3 (17.64)
*Burkholderia cepacia* complex, n (%)	8 (11.94)	4 (12.5)	3 (17.64)
*Escherichia coli*, n (%)	8 (11.94)	5 (15.62)	2 (11.76)
Enterobacter spp., n (%)	3 (4.47)	2 (6.25)	1 (5.88)
Others, n (%)	19 (28.35)	8 (25)	3 (17.64)
Total	67	32	17

**Table 2:** Distribution of MDR and XDR Gram-positive cocci. MDR: multidrug-resistant; XDR: extensively drug-resistant

Organism	Total (n=33)	MDR (n=23)	XDR (n=2)
*Staphylococcus haemolyticus*, n (%)	6 (18.18)	4 (17.39)	1 (50)
*Staphylococcus epidermidis*, n (%)	2 (6.06)	2 (8.69)	0
*Staphylococcus capitis*, n (%)	1 (3.03)	1 (4.34)	0
*Staphylococcus hominis*, n (%)	1 (3.03)	0	0
*Staphylococcus aureus*, n (%)	13 (39.39)	9 (39.13)	1 (50)
Enterococcus spp., n (%)	7 (21.21)	6 (26.08)	0
*Streptococcus agalactiae*, n (%)	1 (3.03)	0	0
Other Gram-positive bacteria, n (%)	2 (6.06)	1 (4.34)	0
Total	33	23	2

Among CoNS, *Staphylococcus haemolyticus* (6, 18.2% of total Gram-positive cocci) was the most common species. A high proportion of Gram-positive isolates exhibited multidrug resistance (23, 69.7%). Among these MDR strains, *S. aureus* (9, 39.1% of MDR Gram-positive cocci) was again the most frequent. Only two extensively drug-resistant (XDR) Gram-positive isolates were identified, one *S. aureus* and one *S. haemolyticus.* The overall and stratified antibiotic resistance patterns are detailed in Table 3. Among all bacterial isolates, high baseline resistance was observed to fluoroquinolones (60.2%), carbapenems (50.8%), and third-generation cephalosporins (63.6% in Gram-negative bacteria). Resistance rates were markedly higher in MDR and XDR subsets. For XDR isolates, resistance exceeded 80% for fluoroquinolones (83.3%) and carbapenems (86.7%), and was nearly universal for piperacillin-tazobactam (92.9%) and cephalosporins (94.1%) among Gram-negative bacteria.

**Table 3:** Antimicrobial resistance patterns among total MDR and XDR bacterial isolates. Antimicrobial resistance patterns among bacterial isolates (total n=100), including Gram-negative bacteria (GNB, n=67) and Gram-positive bacteria (GPB, n=33). Values are expressed as n/N (%), where n is the number of resistant isolates, and N is the number of isolates tested against the respective antimicrobial class. The total number of multidrug-resistant (MDR) isolates was 55, and the number of extensively drug-resistant (XDR) isolates was 19. Denominators vary across antimicrobial classes due to differences in susceptibility testing.

Antimicrobial class	Overall resistance, n/N (%)	MDR isolates, n/N (%)	XDR isolates, n/N (%)
Aminoglycosides (GNB+GPC)	33/93 (35.5)	19/51 (37.3)	13/19 (68.4)
Fluoroquinolones (GNB+GPC)	59/98 (60.2)	40/55 (72.7)	15/18 (83.3)
Carbapenems (GNB+GPC)	30/59 (50.8)	18/29 (62.1)	13/15 (86.7)
Trimethoprim/sulfamethoxazole (GNB+GPC)	29/86 (33.7)	15/52 (28.8)	13/17 (76.5)
Cephalosporins (GNB only)	42/66 (63.6)	23/31 (74.2)	16/17 (94.1)
Piperacillin-tazobactam (GNB only)	35/56 (62.5)	22/28 (78.6)	13/14 (92.9)
Linezolid (GPC only)	4/30 (13.3)	2/24 (8.3)	1/1 (100)
Tetracycline (GPC only)	12/35 (34.3)	7/25 (28.0)	2/2 (100)

## Discussion

This study from a tertiary NICU in Eastern India reveals a disturbing predominance of Gram-negative bacteria (67%), primarily *K. pneumoniae* and Acinetobacter spp., in neonatal sepsis, aligning with recent national and global trends [15-17]. The high prevalence of MDR (55%) and XDR (19%) phenotypes is a critical finding, underscoring the severe AMR crisis in neonatal care.

The leading role of *K. pneumoniae* as an MDR and XDR pathogen is consistent with reports from other Indian studies [18,19]. Its success is attributed to its genomic plasticity, which enables the acquisition of resistance determinants such as extended-spectrum beta-lactamase (ESBL) and carbapenemase genes (e.g., NDM, OXA-48-like) via mobile genetic elements [20]. Similarly, *A. baumannii*, known for its environmental resilience, exhibited high XDR rates, mirroring findings from other high-burden settings [21].

The bacteriological profile observed in our cohort is consistent with the evolving epidemiology of neonatal sepsis across various geographic settings. A recent 2023 multi-centric study from India reported a similar predominance of Gram-negative organisms (68%), with *Klebsiella pneumoniae *and Acinetobacter spp. being the leading causes of culture-positive sepsis and mortality [22]. This convergence of findings across different regional NICUs suggests a potential national shift in the etiology of neonatal sepsis, moving away from the historically common Group B Streptococcus and *Staphylococcus aureus* seen in high-income countries. International data from low- and middle-income countries (LMICs) corroborate this trend; for instance, a 2024 systematic review of neonatal sepsis in South Asia and Sub-Saharan Africa identified *K. pneumoniae* as the most frequently isolated pathogen, often carrying high resistance rates to commonly used empirical antibiotics [23]. This underscores that our findings are not isolated but rather part of a broader public health challenge affecting resource-limited settings globally. The similarity in pathogen profiles across these diverse geographies points to common drivers of resistance, such as widespread over-the-counter antibiotic use and challenges in infection control.

The antimicrobial resistance landscape has continued to evolve, with reports indicating an escalation in resistance even against newer antimicrobial agents. While our study documented high carbapenem resistance, recent surveillance data from 2024 and 2025 suggest further proliferation of metallo-beta-lactamases (such as NDM) and oxacillinases (OXA-48-like) among Enterobacteriaceae in Indian NICUs, leading to increased reliance on last-resort combinations [24]. The carbapenem-sparing strategy, which we noted as potentially flawed given high baseline resistance to piperacillin-tazobactam, is being re-evaluated in light of emerging resistance to newer beta-lactam/beta-lactamase inhibitor combinations. For example, while ceftazidime-avibactam has been a breakthrough for some carbapenem-resistant infections, reports of emerging resistance, particularly in *K. pneumoniae* strains co-harboring multiple resistance mechanisms, are now appearing in the neonatal population [25]. Furthermore, the global dissemination of highly resistant *K. pneumoniae* sequence types (e.g., ST231 and ST147) has been documented, which are often associated with worse outcomes [26]. These recent developments highlight the dynamic nature of AMR and reinforce the critical need for continuous microbiological surveillance and molecular characterization of resistance genes.

The significantly higher mortality in XDR sepsis (31.6%) compared to MDR sepsis highlights the dire clinical consequences of extreme drug resistance, a finding corroborated by international data [27]. This mortality, though high, was lower than in some reports, possibly due to early sepsis recognition, vigorous supportive care, and empirical antibiotic choices guided by local antibiograms [28].

The high resistance to piperacillin-tazobactam (92.9% in XDR) and cephalosporins (94.1%) likely stems from their extensive use as first-line empirical therapy in our unit, a practice common in many Indian NICUs [29]. This creates a vicious cycle of resistance, emphasizing the flawed "carbapenem-sparing" strategy when the baseline resistance to broader-spectrum beta-lactams is already high [30].

Our findings align with a 2023 Indian multi-centric study and 2024 global LMIC data, confirming a shift toward GNB-led sepsis [18,19]. This convergence highlights a shared public health challenge driven by over-the-counter antibiotic use and infection control gaps; moreover, it reinforces the World Health Organization's classification of ESBL-producing *K. pneumoniae* and carbapenem-resistant *A. baumannii* as critical-priority pathogens [31]. The emergence of XDR strains signals a move closer to the nightmare of pan-drug-resistant (PDR) neonatal sepsis. To mitigate this, empirical therapy must be guided by this local data through robust antibiotic stewardship programs that enforce restriction policies, promote shorter courses, and audit practices [32]. Given the alarming resistance rates to conventional first-line therapy (piperacillin-tazobactam >90% in XDR isolates), relying on these agents as a default empirical strategy carries a high risk of initial treatment failure. Clinicians must shift toward a more dynamic approach as follows: prioritizing rapid escalation to appropriate second-line or combination therapy upon the first sign of clinical deterioration in high-risk patients.

This urgent threat necessitates an immediate, multipronged response. Continuous, unit-specific surveillance of bacteriological profiles and resistance patterns is essential. Simultaneously, strict infection prevention and control measures, including hand hygiene and contact precautions, are vital to break transmission chains. Investment in rapid diagnostic tests can facilitate swift, targeted therapy [33]. Finally, multicentric research incorporating advanced molecular techniques is needed to understand the transmission dynamics and resistance mechanisms of these high-risk clones [34]. Without such decisive and coordinated action, the prospect of effectively managing neonatal sepsis will become increasingly bleak.

Limitations

While this single-center study provides valuable local data, it highlights the need for multi-center research to enhance generalizability across diverse NICU settings. The limited molecular characterization of resistance genes points toward an important avenue for future studies to explore genetic mechanisms more comprehensively. Potential biases in empirical treatment before culture underscore the importance of prospective stewardship interventions. The absence of long-term outcome data beyond discharge presents an opportunity for follow-up studies to assess the lasting impact of MDR/XDR neonatal sepsis.

## Conclusions

This study reveals a high burden of MDR and XDR Gram-negative neonatal sepsis in our setting, predominantly driven by *Klebsiella pneumoniae* and *Acinetobacter baumannii*. The high resistance observed to carbapenems and other last-resort antibiotics highlights the increasing complexity of managing neonatal infections. This is essential for optimizing empirical therapy, improving clinical outcomes, and preventing the further emergence of pan-drug-resistant strains. Our findings underscore that addressing this challenge requires a coordinated, evidence-based approach to preserve the efficacy of current and future antimicrobial options.
